# Flexible Sensor with Material–Microstructure Synergistic Optimization for Wearable Physiological Monitoring

**DOI:** 10.3390/ma18153707

**Published:** 2025-08-07

**Authors:** Yaojia Mou, Cong Wang, Xiaohu Jiang, Jingxiang Wang, Changchao Zhang, Linpeng Liu, Ji’an Duan

**Affiliations:** 1 State Key Laboratory of Precision Manufacturing for Extreme Service Performance, College of Mechanical and Electrical Engineering, Central South University, Changsha 410083, China; 233712156@csu.edu.cn (Y.M.); wangcong@csu.edu.cn (C.W.); duanjian@csu.edu.cn (J.D.); 2 College of Mechanical and Electrical Engineering, Hunan Agricultural University, Changsha 410128, China; 18844198030@163.com; 3 College of Engineering Technology, Jilin Agricultural University, Changchun 130118, China; jingxiangwang@jlau.edu.cn; 4 Key Laboratory of Bionic Engineering Ministry of Education, Jilin University, Changchun 130022, China; changchaozhang@jlu.edu.cn

**Keywords:** flexible sensor, bilayer electrode, material optimization, laser direct writing, gradient crack microstructure

## Abstract

Flexible sensors have emerged as essential components in next-generation technologies such as wearable electronics, smart healthcare, soft robotics, and human–machine interfaces, owing to their outstanding mechanical flexibility and multifunctional sensing capabilities. Despite significant advancements, challenges such as the trade-off between sensitivity and detection range, and poor signal stability under cyclic deformation remain unresolved. To overcome the aforementioned limitations, this work introduces a high-performance soft sensor featuring a dual-layered electrode system, comprising silver nanoparticles (AgNPs) and a composite of multi-walled carbon nanotubes (MWCNTs) with carbon black (CB), coupled with a laser-engraved crack-gradient microstructure. This structural strategy facilitates progressive crack formation under applied strain, thereby achieving enhanced sensitivity (1.56 kPa^−1^), broad operational bandwidth (50–600 Hz), fine frequency resolution (0.5 Hz), and a rapid signal response. The synergistic structure also improves signal repeatability, durability, and noise immunity. The sensor demonstrates strong applicability in health monitoring, motion tracking, and intelligent interfaces, offering a promising pathway for reliable, multifunctional sensing in wearable health monitoring, motion tracking, and soft robotic systems.

## 1. Introduction

With the rapid advancement of emerging technologies such as wearable electronics, smart healthcare, soft robotics, and human–machine interfaces, flexible sensors have emerged as a critical enabling component that integrates mechanical compliance with multifunctional sensing capabilities [1,2,3,4,5], thereby offering broad prospects for future technological development. Their key advantages, including superior flexibility, light weight, stretchability, and conformal integration with complex surfaces [6,7,8,9], allow flexible sensors to operate reliably in harsh and dynamic environments where conventional rigid sensors often fail. As a result, they have demonstrated significant potential for application in cutting-edge fields such as health monitoring [10,11], structural diagnostics [12,13,14], human–machine interaction [15], intelligent wearable devices [16,17], electronic skin [18], robotic tactile systems [19,20], and environmental surveillance [21]. In the domain of physiological monitoring, flexible sensors are capable of continuously acquiring vital signs, such as pulse, respiration, body temperature, electromyography (EMG), and blood oxygen levels, providing reliable support for disease prevention, remote diagnosis, and personalized medicine [22,23,24]. In motion detection and wearable systems, they can detect subtle body movements, postural shifts, and fatigue states, thus supporting the development of smart clothing, interactive insoles, and virtual reality platforms [25,26,27,28]. In the fields of electronic skin and bioinspired robotics, flexible sensors mimic the sensory functions of human skin, enabling the detection of pressure, strain, temperature, and humidity and endowing robots with environmental perception and adaptive behavior [29,30,31,32,33]. Furthermore, in areas such as industrial structure monitoring, soft interface sensing, and intelligent control systems, flexible sensors offer distinct advantages, including high sensitivity, multi-modal signal compatibility, and low power consumption [34,35,36,37,38,39]. Through optimized material [40,41,42] and structural design [43,44], flexible sensors can also achieve a desirable balance among high sensitivity, broad detection range, fast response, and long-term durability, which enables stable operation under continuous mechanical deformation. These collective merits position flexible sensors as a pivotal technology in next-generation multifunctional intelligent systems and a foundational enabler for achieving the vision of ubiquitous connectivity and intelligent perception.

However, current flexible sensors continue to face the critical challenge of achieving a balance between sensitivity and sensing range [45]. Due to the limitations imposed by substrate and electrode materials, many conventional flexible sensors exhibit poor signal stability, limited repeatability, and inconsistent electrode performance, particularly under prolonged or repeated mechanical deformation. At present, two major approaches are commonly employed to enhance sensor performance: material optimization and structural design enhancement. Du et al. [46] developed a scalable and straightforward fabrication approach for flexible pressure sensors by integrating silver nanowire (AgNW) coatings, laser-ablated hierarchical microstructured PDMS, and interdigitated electrodes. This design achieved a high sensitivity of 4.48 kPa^−1^ and a sensing range ranging from 0 to 65 kPa. The hierarchical microstructure increased the effective contact area, which in turn enhanced the sensor’s responsiveness. When applied to the human body, the sensor successfully captured physiological signals such as arterial pulse, respiration, and static tremor, demonstrating the effectiveness of structural optimization in enhancing sensor performance. From the perspective of material engineering, Yang et al. [47] reported a microstructured pressure sensor based on a composite of reduced graphene oxide (rGO), carbon nanotubes (CNTs), and PDMS. The pressure sensor demonstrated notable responsiveness, achieving a sensitivity of 0.2 kPa^−1^ under low-pressure conditions (0–13 kPa), while also supporting a broad sensing range up to 180 kPa. Moreover, the device was successfully applied to handwriting recognition and plantar pressure mapping, highlighting its multifunctional capabilities and the effectiveness of material-level engineering strategies in flexible sensor design. Moreover, Antonio del Bosque et al. [48] developed a highly stretchable strain sensor by reinforcing Ecoflex silicone elastomer with carbon nanotubes (CNTs), aiming at applications in respiratory signal tracking. Strain response tests under both tensile and compressive conditions demonstrated a broad detection range, along with ultrahigh sensitivity at elevated strain levels. Specifically, a nominal gauge factor (GF) of approximately 10^4^ was recorded at a CNT loading of 0.3 wt%, which further increased to 10^5^ at a strain level of 300%. The sensor exhibited stable electrical performance under 2000 cyclic loading tests and across varying frequencies. In addition, the team integrated this sensor into a wireless respiration monitoring platform using low-power Bluetooth technology. This system was capable of acquiring, filtering, visualizing, and storing breathing signals in real-time, enabling the accurate identification of respiratory rate and inspiration–expiration differences. Such an innovative solution highlights the practical potential of materials-level optimized sensors in high-impact scenarios, including clinical diagnostics, emergency rescue, and first-aid respiratory monitoring.

Building upon the integrated optimization of materials and microstructure, this study presents a high-performance flexible sensor featuring a bilayer electrode and a laser-engraved gradient crack microstructure. The gradient in crack width enables gradual and controllable crack propagation during strain loading [49], thereby providing the sensor with high sensitivity to subtle deformations, a broad detection range, and excellent signal resolution. The bilayer electrode—comprising a conductive AgNP top layer and MWCNT/CB composite—ensures both signal integrity and mechanical robustness [50,51]. This synergistic configuration effectively improves signal repeatability, noise immunity, and long-term operational stability, highlighting its strong potential for high-resolution and reliable sensing applications in wearable electronics and soft systems. Experimental results further validate the effectiveness of this approach. The sensor demonstrates balanced performance, exhibiting a sensitivity of 1.56 kPa^−1^ while accurately responding to vibration signals across a wide frequency range (50–600 Hz). In addition, the device demonstrates precise waveform recognition, a fine frequency resolution of 0.5 Hz, and responds swiftly, thereby underscoring its wide application potential and practical significance in the field of flexible sensing technologies.

## 2. Materials and Methods

### 2.1. Materials

The Polydimethylsiloxane (PDMS, Sylgard 184) utilized in this work was sourced from Dow Corning (Midland, MI, USA). Multi-walled carbon nanotubes (MWCNTs, 10–20 nm in diameter and 10–30 µm in length) were obtained from Nanjing XFNANO Materials Tech Co., Ltd. (Nanjing, China). Ethyl acetate, serving as the solvent, was supplied by Sinopharm Chemical Reagent Co., Ltd. (Shanghai, China). Carbon black (model ECP-600JD) was obtained from Tianjin Aiweixin Chemical Technology Co., Ltd. (Tianjin, China), and the silver sputtering source was supplied by Zhengzhou Ketan Instrument Equipment Co., Ltd. (Zhengzhou, China). The epoxy adhesive used in this study was sourced from Ausbond Co., Ltd. (Shenzhen, China), while the silver conductive paste (JY12) was acquired from Shanghai Julong Electronic Technology Co., Ltd. (Shanghai, China). Copper paste came from Dongguan Hengchuang Adhesive Products Co., Ltd. (Dongguan, China).

### 2.2. Fabrication of the Sensing Layer

#### 2.2.1. Fabrication of the Gradient Crack Microstructure

To construct the gradient-width crack microstructure, a femtosecond laser (HR-Platform-0203, Huaray Precision Laser, Wuhan, China) was employed to ablate a predesigned pattern onto an acrylic substrate. The pattern comprised crack-like channels with a constant length of 15 mm, spacing of 4 mm, and gradually decreasing widths of 800 μm, 700 μm, 600 μm, 500 μm, and 400 μm. The laser system functioned at a wavelength of 1030 nm, utilizing a scan rate of 100 mm/s, with 75% output power and a pulse frequency of 1000 Hz. A galvanometer scanner (Scanlab, Puchheim, Germany) was used to perform 20 repeated scans per region, ensuring a uniform ablation depth of approximately 350 μm across all features. Post-ablation, the acrylic substrate was cleaned ultrasonically in deionized water to remove any residual debris and oxidation.

A two-part epoxy system was mixed in a 2:1 weight ratio and stirred until uniform and bubble-free. The mixture was then poured into the laser-ablated mold. After curing at room temperature for 1 h, a negative mold containing the crack structure was obtained. Subsequently, the degassed PDMS was poured into the epoxy mold and thermally cured in an oven at 70 °C for 1 h. The fabricated PDMS film, measuring 450 μm in thickness, 15 mm in width, and 30 mm in length, accurately replicated the intended crack pattern with a gradient in width.

#### 2.2.2. Construction of the Bilayer Electrode

The bilayer electrode was fabricated by first preparing a conductive ink. The PDMS prepolymer was formed by mixing the base and curing agent (10:1 by weight). This was followed by adding MWCNTs, CB, PDMS, and EtOAc in a 1:5:30:500 weight ratio. The solution was magnetically agitated at 500 rpm for half an hour, followed by 5 min of ultrasonic treatment to promote homogeneous dispersion.

The resulting ink was deposited onto the PDMS surface using air-assisted spray coating over the predefined conductive regions. Then the coated film was cured at 100 °C for 1 h, allowing solvent evaporation and PDMS crosslinking. A shadow mask was aligned to the coated side, and a 300 nm thick silver layer was deposited using magnetron sputtering under a 30 mA current to form the top electrode, completing the bilayer architecture.

#### 2.2.3. Sensor Integration

Copper foil strips were attached to each end of the electrode region after coating the contact areas with silver paste to establish conductive terminals. To guarantee reliable electrical contact, the integrated device underwent thermal curing at 120 °C for 1 h under reduced pressure using a vacuum oven. External wires were then soldered to the copper foils, finalizing the sensor assembly process.

## 3. Results and Discussion

**Figure 1:**
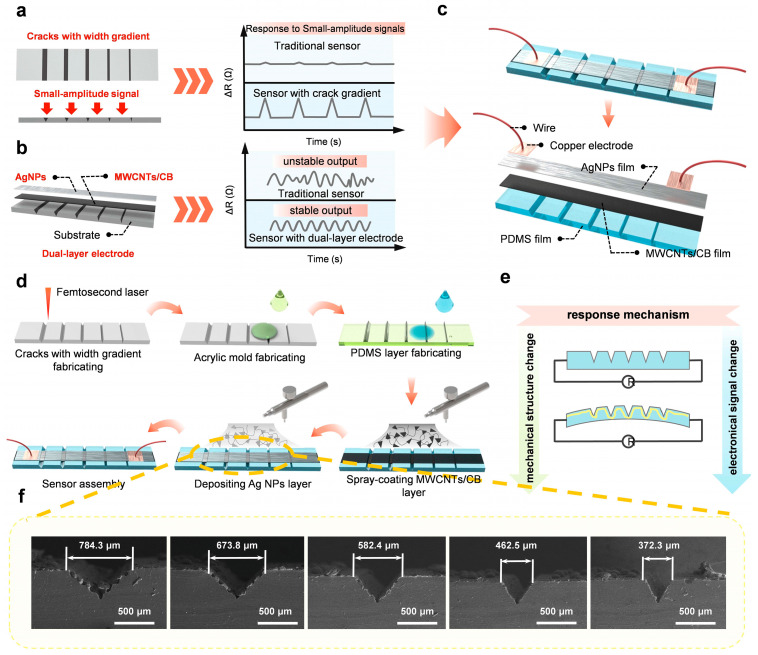
Diagram and manufacturing procedure of the vibration sensor. (**a**) Optimization of the crack microstructure with a gradient in width. (**b**) Optimization of the bilayer electrode structure. (**c**) Overall sensor model and exploded view. (**d**) Manufacturing procedure of the vibration sensor. (**e**) Schematic illustration of the sensor’s working mechanism. (**f**) SEM image of crack structures with a width gradient.

Figure 1a,b offers a clear depiction of the multi-level optimization strategy employed in this study for flexible sensor design. From a structural perspective, a crack microstructure with a gradient in width was implemented. Under applied strain, this gradient structure facilitates sequential crack propagation from shallow to deep regions, resulting in finely tunable resistance variations. This “layer-by-layer activation” mechanism significantly enhances the sensor’s sensitivity to subtle deformations and improves signal resolution. For the purpose of optimizing materials, a dual-layer electrode structure featuring synergistic functions was utilized. This configuration consisted of an AgNP layer and a lower composite layer composed of MWCNTs blended with CB. The AgNP top electrode offers excellent electron mobility and low contact resistance. The nanoscale particles form face-to-face contact interfaces that ensure stable electrical conduction even under strain, thereby contributing to enhanced device sensitivity. The MWCNT/CB bottom electrode combines the superior longitudinal conductivity and tensile flexibility of MWCNTs with the high interfacial adhesion and structural stability provided by carbon black. Together, they form a robust three-dimensional conductive network that effectively suppresses electrode cracking and signal drift caused by repeated mechanical deformation.

By integrating these two optimization strategies, the sensor shown in Figure 1c was developed. The device comprises a PDMS substrate layer, an MWCNT/CB composite electrode layer, a top AgNP electrode layer, and copper electrodes with external wiring. The fabrication process is illustrated in detail in Figure 1d. The working mechanism of the sensor is illustrated schematically in Figure 1e. At the macroscopic level, mechanical loading induces deformation of the flexible PDMS layer, which alters the resistance of the conductive pathway and converts mechanical stimuli into electrical signals. Mechanical deformation at the microscale leads to stretching or partial breakage of the conductive network formed by MWCNTs and carbon black. This effect reduces the quantity of conductive channels, resulting in an increase in the sensor’s total electrical resistance. Consequently, this mechanism facilitates the conversion of mechanical forces into measurable electrical signals. Figure 1f presents the SEM image of the engineered crack microstructures in the fabricated sensor. As clearly observed, the crack arrays, generated via the femtosecond laser-assisted process, exhibit the intended gradient in crack width, with uniform alignment and well-defined morphology, demonstrating excellent spatial consistency and structural controllability. Moreover, a quantitative cross-sectional analysis was conducted on multiple cracks, revealing that the crack depths were consistently distributed within the range of 325–350 μm, with minimal variation. These findings underscore the high reproducibility and precision of the proposed fabrication method, further confirming the engineering feasibility of the designed sensor architecture.

**Figure 2:**
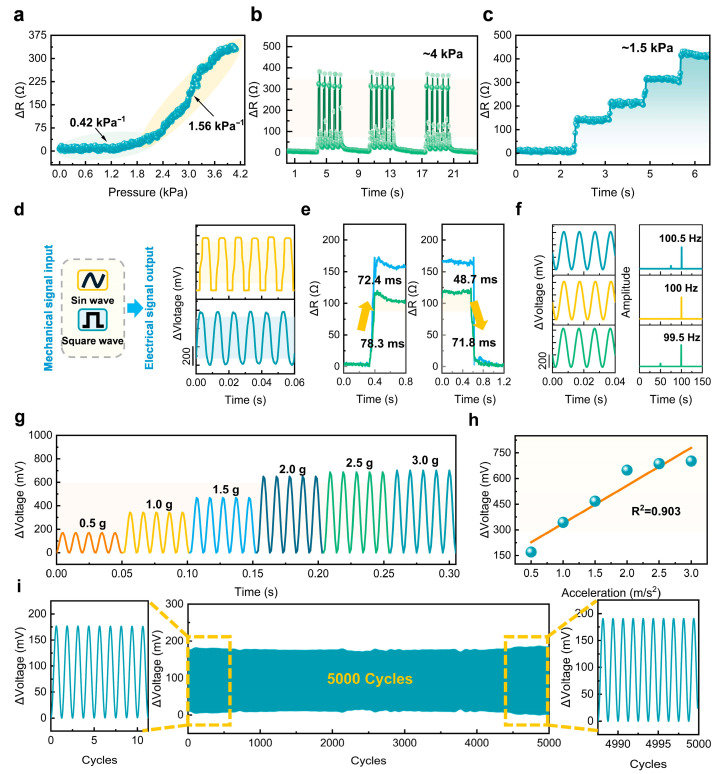
Evaluation of the developed vibration sensor’s signal response and functional performance. (**a**) Relative resistance changes under applied pressure within the range of 0 to 4.2 kPa. (**b**) Resistance variation under cyclic loading at 4 kPa. (**c**) Resistance response to stepwise increasing pressure up to 1.5 kPa. (**d**) Sensor responses to 100 Hz vibrations with different waveforms (square wave and sine wave). (**e**) Response and recovery times under different applied loads. (**f**) Resolution test results of the sensor. (**g**) Sensor output under 100 Hz vibrations with varying acceleration amplitudes. (**h**) Results of sensor linearity characterization. (**i**) Electrical stability of the developed sensor under 5000 cyclic loading conditions.

To evaluate the fundamental sensing behavior of the fabricated device, a set of standardized experiments was carried out. The sensitivity was determined using the gauge coefficient (GF), which is expressed as GF = (ΔR/R_0_)/ΔP. Here, ΔR indicates the change in resistance (ΔR = R − R_0_), where R_0_ is the reference resistance and R represents the instantaneous measured value. The applied load variation is denoted as ΔP. This metric effectively characterizes the sensor’s response under varying mechanical input. During the test, a pressure range of 0 to 4.2 kPa was applied by coupling a load cell with a precision-controlled stepper motion stage. As shown in Figure 2a, the sensor exhibited two distinct sensitivity regimes: in the low-pressure range (0–2.4 kPa), the sensitivity was GF_1_ = 0.42 kPa^−1^, and in the high-pressure range (2.4–4.2 kPa), it increased to GF_2_ = 1.56 kPa^−1^. For stability evaluation, cyclic loading tests were performed at a constant pressure of 4 kPa. The sensor demonstrated excellent signal consistency and repeatability (Figure 2b).

Under four sequential 1.5 kPa step loads (Figure 2c), the sensor produced well-defined, stepwise responses, confirming its capability for step recognition. The waveform resolution was assessed using 100 Hz square and sine wave inputs (Figure 2d), where the sensor accurately reproduced the input signals, demonstrating high waveform fidelity. The dynamic behavior of the sensor was further evaluated by measuring its response and recovery durations under varying pressure conditions. When subjected to higher pressure, the sensor’s reaction and relaxation durations were recorded as 72.4 ms and 48.7 ms, respectively. In contrast, under reduced loading conditions, these intervals increased slightly to 78.3 ms and 71.8 ms (Figure 2e). These results demonstrate the sensor’s fast real-time responsiveness to external mechanical perturbations. To further evaluate resolution and linearity, vibration tests were conducted using a vibration excitation system and power amplifier. The sensor was subjected to closely spaced frequencies of 99.5 Hz, 100 Hz, and 100.5 Hz. FFT analysis (Figure 2f) revealed well-separated peaks that precisely corresponded to the input frequencies, indicating a frequency resolution of 0.5 Hz. The sensor was then subjected to a fixed 100 Hz frequency with variable accelerations ranging from 0.5 g to 3.0 g. As illustrated in Figure 2g, the electrical output exhibited proportionality to the applied acceleration, and a linear fit yielded a correlation coefficient of 0.903 (Figure 2h). Furthermore, the long-term stability of the developed sensor was evaluated by subjecting it to continuous cyclic loading under a sinusoidal excitation at 100 Hz. As illustrated in Figure 2i, the sensor maintained excellent electrical stability over 500 consecutive loading cycles, with no noticeable signal drift observed throughout the test. These results provide compelling evidence of the sensor’s robustness and durability, highlighting its potential for long-term operation and repeated use. This also further validates the effectiveness and practicality of the material and structural optimization strategies proposed in this study. These results collectively demonstrate that the proposed sensor achieves a favorable balance between high sensitivity and operational stability, while also exhibiting excellent resolution and linearity, thereby validating the effectiveness and practical applicability of the integrated optimization strategy.

**Figure 3:**
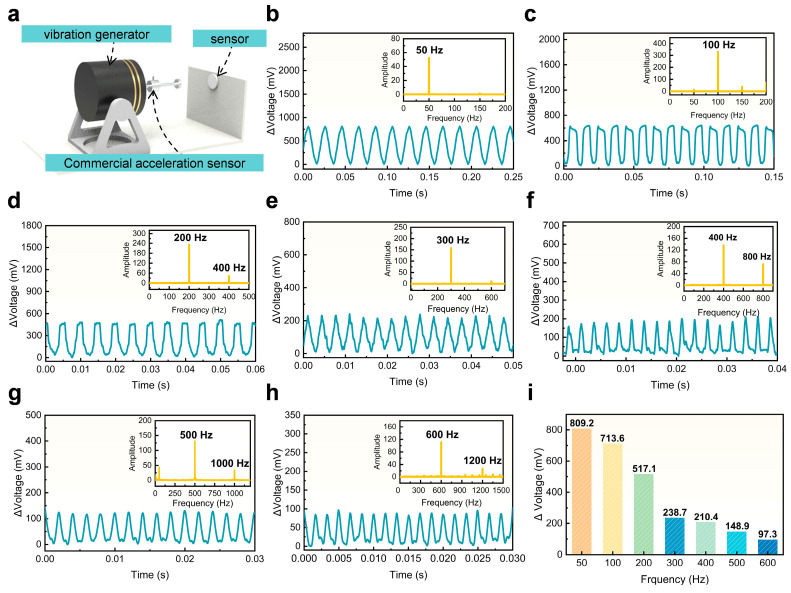
Operating bandwidth of the vibration sensor. (**a**) Illustration of the experimental setup used for vibration frequency characterization. (**b**–**h**) Time-domain signals and corresponding frequency-domain spectra (via FFT) obtained under periodic mechanical excitations with constant frequencies ranging from 50 Hz to 600 Hz. (**i**) Summary comparison of output signal amplitudes across varying excitation frequencies.

To further validate the effectiveness of the proposed hierarchical material-level optimization strategy in improving sensor performance, a high-precision dynamic testing platform was constructed, as illustrated in Figure 3a, to evaluate the sensor’s responsiveness to high-frequency, low-amplitude vibration signals. This testing system consisted of a function generator and power amplifier-driven electromagnetic vibration exciter, along with a commercial high-sensitivity accelerometer, enabling the application of precisely controlled periodic mechanical stimuli.

Figure 3b–h display the sensor’s real-time responses under mechanical stimuli at discrete frequencies of 50 Hz, 100 Hz, 200 Hz, 300 Hz, 400 Hz, 500 Hz, and 600 Hz. The measured signals exhibit strong temporal agreement with the applied vibrations, demonstrating high fidelity and signal clarity. To further verify the frequency correspondence, Fast Fourier Transform (FFT) was performed on the recorded outputs. The resulting spectra show distinct peaks corresponding precisely to the input frequencies under all excitation conditions, confirming the sensor’s stable and accurate frequency response. Additionally, as illustrated in Figure 3i, the amplitude of the sensor output systematically decreases with increasing frequency, a trend consistent with typical frequency response behavior in flexible sensors, further supporting the reliability of the measured signals. Collectively, these results demonstrate that the proposed hierarchical structural design significantly enhances the sensor’s capacity to detect subtle, high-frequency mechanical stimuli, thereby validating both the scientific rationale and practical applicability of the design approach.

**Figure 4:**
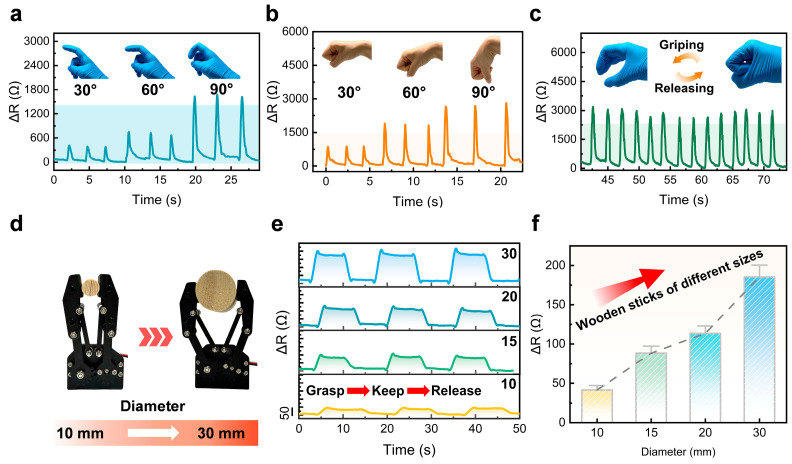
Sensor evaluation under practical conditions. (**a**) Electrical output signals of the sensor during finger bending at varying angles. (**b**) Electrical output signals of the sensor during wrist bending at varying angles. (**c**) Reproducible signal responses of the sensor during cyclic fist clenching. (**d**) Schematic diagram of the robotic grasping experiment using wooden rods. (**e**) Electrical responses of the sensor when grasping wooden rods of different diameters. (**f**) Comparative analysis of the peak resistance values corresponding to different object sizes.

Following the dynamic evaluations, the bilayer electrode structure was further evaluated in real-world application scenarios to verify its enhanced mechanical flexibility and conformability. The fabricated sensor was first affixed to various human joints to evaluate its motion-sensing performance under real-time physiological motion (Figure 4a–c). When mounted on the finger and wrist joints and subjected to incremental bending at 30°, 60°, and 90°, the sensor exhibited synchronous resistance variations, with signal amplitudes increasing proportionally to the bending angle (Figure 4a,b), indicating excellent resolution and high repeatability in capturing joint movements. Furthermore, during rhythmic fist clenching with the sensor adhered to the dorsal hand surface, the resistance exhibited consistent and distinguishable patterns across cycles, highlighting the device’s reliability in dynamic monitoring conditions. Beyond wearable motion tracking, the sensor’s capability for tactile interaction sensing was further demonstrated through robotic grasping experiments. As shown in Figure 4d, the sensor was integrated at the tip of a robotic gripper and accurately captured resistance variations throughout the grasp–hold–release cycle when interacting with wooden rods of varying diameters. The corresponding outputs (Figure 4e) accurately captured each stage of the mechanical interaction, while the peak signal amplitudes (Figure 4f) varied distinctly with object size, demonstrating strong signal discrimination and high measurement stability. These application-driven validations underscore the multifunctionality and practical relevance of the proposed design, which not only enhances adaptability to complex surfaces but also significantly improves responsiveness to subtle dynamic stimuli, positioning it as a promising candidate for next-generation wearable electronics, tactile perception, and human–machine interface systems.

## 4. Conclusions

To summarize, this work demonstrates a flexible sensor designed through the coordinated integration of a dual-layer electrode (AgNP/MWCNT–CB) and a laser-engraved gradient crack microstructure. This integrated configuration significantly enhances the sensor’s capability to detect subtle deformations, broadens the effective sensing range, and offers improved mechanical flexibility and enhanced signal stability. Experimental evaluations demonstrate that the sensor achieves a sensitivity of 1.56 kPa^−1^, a wide frequency response range from 50 to 600 Hz, a frequency resolution of 0.5 Hz, excellent waveform recognition, rapid response, and high repeatability. Collectively, these results underscore its practical potential in wearable sensing, dynamic monitoring, and intelligent human–machine interfaces.

## Data Availability

The original contributions presented in the study are included in the article, further inquiries can be directed to the corresponding author.
